# Laparoscopic Bipolar Coagulation of Hypogastric Artery in Postpartum Haemorrhage: A Case Report

**DOI:** 10.1155/2011/250325

**Published:** 2011-09-06

**Authors:** Enrico Panuccio, Eugenio Volpi, Annamaria Ferrero, Piero Sismondi

**Affiliations:** Academic Department of Gynaecological Oncology, Mauriziano Umberto I Hospital, Largo Turati 62, 10128 Turin, Italy

## Abstract

*Background*. Postpartum haemorrhage (PPH) is a significant contributor to worldwide maternal morbidity and mortality. When PPH continues despite aggressive medical treatment, early consideration should be given to surgical intervention. Various surgical interventions may be used but conservative interventions are recommended primarily. *Case*. This case report describes laparoscopic coagulation of hypogastric artery technique in a patient with PPH. *Conclusions*. Laparoscopic ligature of the hypogastric artery for PPH treatment can be a valid alternative to laparotomy in patients with vaginal delivery.

## 1. Introduction

Death as a consequence of pregnancy remains an important cause of premature mortality worldwide. An estimated 500,000 women die from this potentially preventable cause each year [1], with up to an estimated quarter of these deaths occurring as a consequence of haemorrhage. Although the majority of these deaths occur in low income countries, several recent publications [2] have noted an increasing trend in the incidence of postpartum haemorrhage (PPH) over time in high resource countries.

Causes of PPH can be uterine (atony, retained placental products, abnormal placentation, lacerations, uterine inversion, and rupture) or nonuterine (lower genital tract lacerations, coagulopathy). More than 90% of PPH is uterine related.

The management of PPH includes a stepwise approach which starts with the exclusion of retained products and genital tract trauma. Uterine atony, which is the most common cause, is dealt with uterine rubbing and various uterotonic drugs.

When PPH continues despite aggressive medical treatment, early consideration should be given to surgical intervention [3].

The aim of this case report is to describe our recent experience with laparoscopic coagulation of hypogastric artery in a woman with PPH after vaginal delivery.

## 2. Patient

A 44-year-old woman in her second pregnancy had a spontaneous vaginal delivery of a male infant weighting 3480 g with 1 and 5 minutes Apgar scores of 9 and 10. At admission haemogloblin level (Hb) was 11.6 g/dL, platelets count 252.000 u.l, aPTT 35.8 sec, Pt/ratio 1.05 and fibrinogen 376 mg/dL.

Vaginal tract laceration was diagnosed and immediately repaired. Vaginal bleeding estimated at partum was about 1000 mL. For this reason curettage of uterine cavity was performed. Uterotonics drugs were also administered. In consideration of the drop of laboratory data (Hb 6.9 g/dL, platelets count 150.000 u.l., aPTT 30.2 sec, Pt/ratio 1.00 and fibrinogen 285 mg/dL), 3 units of blood were transfused. No intraperitoneal bleeding was shown by ultrasound examination. 

Massive vaginal bleeding recurred after 36 hours. At this time laboratory data were Hb 6.2, platelets 80.000 count, aPTT 31.1, Pt/ratio 0.91, and fibrinogen 238. Initially, uterine curettage was executed, showing abundant leakage of placental material from the uterine cavity. After curettage, a packing of the uterine was performed. Massive vaginal bleeding persisted and therefore laparoscopic bipolar coagulation of hypogastric artery was considered. We did not plan selective arterial embolization in consideration of the time necessary to shuffle the patient to radiologic suite.

## 3. Surgical Technique

At abdominal examination the uterus was 3 cm below the umbilicus. Four ports were placed in the abdomen: a 10-mm port through the umbilicus for videolaparoscope and three 5-mm ports in the usual diamond position. At the pelvic exploration we did not see intraperitoneal haemorrhage or haematomas.

**Figure 1:**
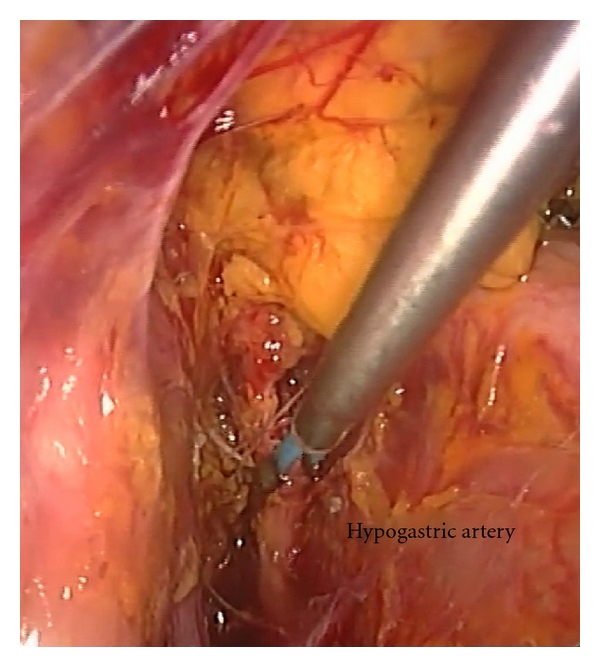
Hypogastric artery was isolated and coagulated with bipolar current.

We used the triangle enclosed by the round ligament, external iliac vessels, and infundibulopelvic ligament for opening the broad ligament. We identified the ureter medial to the internal iliac artery after vertical incision of peritoneum above the psoas muscle. After opening the proximal part of the left pararectal space the hypogastric artery was isolated and coagulated with bipolar current (Figure 1).The same procedure was performed on the right side. Vaginal bleeding considerably decreased after bilaterally hypogastric artery coagulation. The laparoscopic operative time was 30 minutes.

We observed the patient in the surgical theatre for two hours to evaluate the bleeding. During this time we transfused 4 units of blood and 1000 cc of plasma.

After surgical intervention, the patient was transferred in adult intensive care unit; 2 units of blood and 1000 cc of plasma were transfused. Twenty-four hours later the patient was haemodynamically stable and was transferred to our maternity ward. The patient was discharged 7 days after delivery. 

At 12-month followup the patient is asymptomatic for pelvic disease and physiologically menstruating.

## 4. Discussion

The management of PPH involves a stepwise escalation of pharmacological and surgical approaches.

Surgical management of PPH for young women or with low parity is a dilemma for obstetricians. Various surgical interventions may be used. These include internal iliac artery ligation, uterine compression sutures (B-Lynch), uterine balloon tamponade, and peripartum hysterectomy to control the life-threatening haemorrhage [3]. To our knowledge, the laparoscopic coagulation of hypogastric artery has not been reported in the management of PPH. Our case report suggests that laparoscopic coagulation of hypogastric artery can be a possible treatment for PPH. 

The choice of the right procedure depends on several consideration, including hemodynamic status, parity, the desire of childbearing, the extent of haemorrhage, and, most importantly, on the surgeon's experience. In extreme situations, hysterectomy is preferred in order to arrest further blood loss. Although it is a life-saving procedure, for women who need to preserve their reproductive potential it may not be appropriate and conservative interventions are recommended primarily [3, 4].

Uterine tamponade using intrauterine balloons appears to be an effective tool in the management of PPH, being successful in up to 90% of cases [3]. Few studies report difficulties or failures in using the balloons. Some of these “failures” may be interpreted as “complications of placement”. These include obstruction by uterine leiomyoma, unnoticed damage to the balloon, inability to place the balloon due to the presence of a uterine suture, and insufficient insufflation [5]. However this approach cannot prevent persistent bleeding from the lower uterine segment or the cervix. We did not employ this method because in our department we are not confident with balloon use.

Various uterine suture techniques are presently available. The ease of application of such sutures is a major advantage, and fertility is preserved. The obvious disadvantages are the need of laparotomy and usually hysterotomy (although some modified types have avoided the latter surgical step of the procedure), and the literature reported complications [6, 7].

Internal iliac and uterine embolization was successfully employed to control obstetric hemorrhage [3]. The most common reported complications from this procedure are fever, lower extremity ischemia, bladder and rectal wall necrosis, neuropathy, thrombosis, delayed bleeding, and uterine necrosis [8–10].

In addition to selective arterial embolization, occlusive uterine balloon catheters may be placed. Selective arterial embolization is an option in managing PPH if the women is haemodynamically stable. We did not apply this technique because we had not enough time to shuffle the patient in radiologic suite. Bilateral hypogastric (internal iliac) artery ligation and bilateral uterine artery ligation after vaginal delivery or after cesarean section are well-documented vascular occlusive method for treating PPH [10]. Since 90% of blood supply of pregnant uterus comes from uterine artery, ligation of that artery is the most efficient of conservative surgical intervention. Laparotomic internal iliac artery ligation is an effective procedure in PPH treatment and prevention in high-risk women. Unlike other procedures, it can be reliably used in PPH from all causes [10]. However, the procedures are performed through an abdominal incision. This method was reported as a simple, safe, life-saving alternative to hysterectomy, with 85% success rates [4].

Our laparoscopic procedure was based on our experience in treating various kinds of benign and malign gynaecologic diseases with the laparoscopic bipolar coagulation of hypogastric artery.

Laparoscopic knowledge of retroperitoneal anatomy and meticulous standardization of operative technique to isolate the ureter and hypogastric artery are required in advance, and this may be a limit to a wider application of the technique.

In the treatment of bleeding in advanced cervical cancer, the laparoscopic ligature is as effective as the classical laparotomic approach while sparing the patient an unnecessary abdominal incision [11].

Chou et al. [12] described a laparoscopic bipolar coagulation of uterine vessels in a delayed PPH. Coagulation of the uterine artery or the hypogastric artery just proximal to the bifurcation with the uterine artery are similar procedures but the former requires a more difficult dissection and the ureter is nearly.

In our opinion laparoscopic ligature can be a valid alternative to laparotomy in patients with PPH with vaginal delivery as it is as effective but less invasive. 

## 5. Conclusion

Our case report suggests that the laparoscopic bipolar coagulation of hypogastric artery in PPH can be an effective, safe, and minimally invasive technique in managing PPH, sparing the patient an unnecessary laparotomy and hospitalization, is much shorter than with abdominal hypogastric artery ligation. Further studies are needed to prove it on a large scale.
